# A functional map of genomic HIF1α-DNA complexes in the eye lens revealed through multiomics analysis

**DOI:** 10.1186/s12864-021-07795-9

**Published:** 2021-07-03

**Authors:** Joshua Disatham, Lisa Brennan, Daniel Chauss, Jason Kantorow, Behdad Afzali, Marc Kantorow

**Affiliations:** 1 Charles E. Schmidt College of Medicine, Florida Atlantic University, 777 Glades Rd., Boca Raton, FL 33431 USA; 2 Immunoregulation Section, Kidney Diseases Branch, National Institute of Diabetes and Digestive and Kidney Diseases (NIDDK), NIH, Bethesda, MD 20892 USA; 3 University of Florida, Gainesville, FL 32611 USA

**Keywords:** HIF1α, Hypoxia, CUT&RUN, ATAC-seq, RNA-seq, Gene regulation, DNA binding, Chromatin, Transcriptional regulation

## Abstract

**Background:**

During eye lens development the embryonic vasculature regresses leaving the lens without a direct oxygen source. Both embryonically and throughout adult life, the lens contains a decreasing oxygen gradient from the surface to the core that parallels the natural differentiation of immature surface epithelial cells into mature core transparent fiber cells. These properties of the lens suggest a potential role for hypoxia and the master regulator of the hypoxic response, hypoxia-inducible transcription factor 1 (HIF1), in the regulation of genes required for lens fiber cell differentiation, structure and transparency. Here, we employed a multiomics approach combining CUT&RUN, RNA-seq and ATACseq analysis to establish the genomic complement of lens HIF1α binding sites, genes activated or repressed by HIF1α and the chromatin states of HIF1α-regulated genes.

**Results:**

CUT&RUN analysis revealed 8375 HIF1α-DNA binding complexes in the chick lens genome. One thousand one hundred ninety HIF1α-DNA binding complexes were significantly clustered within chromatin accessible regions (χ^2^ test *p* < 1 × 10^− 55^) identified by ATACseq. Formation of the identified HIF1α-DNA complexes paralleled the activation or repression of 526 genes, 116 of which contained HIF1α binding sites within 10kB of the transcription start sites. Some of the identified HIF1α genes have previously established lens functions while others have novel functions never before examined in the lens. GO and pathway analysis of these genes implicate HIF1α in the control of a wide-variety of cellular pathways potentially critical for lens fiber cell formation, structure and function including glycolysis, cell cycle regulation, chromatin remodeling, Notch and Wnt signaling, differentiation, development, and transparency.

**Conclusions:**

These data establish the first functional map of genomic HIF1α-DNA complexes in the eye lens. They identify HIF1α as an important regulator of a wide-variety of genes previously shown to be critical for lens formation and function and they reveal a requirement for HIF1α in the regulation of a wide-variety of genes not yet examined for lens function. They support a requirement for HIF1α in lens fiber cell formation, structure and function and they provide a basis for understanding the potential roles and requirements for HIF1α in the development, structure and function of more complex tissues.

**Supplementary Information:**

The online version contains supplementary material available at 10.1186/s12864-021-07795-9.

## Background

The mature eye lens is composed of an anterior layer of organelle-containing epithelial cells that during development and throughout adult life continuously differentiate to form a core of transparent organelle-free lens fiber cells [1]. The process of mammalian eye lens formation occurs during major changes in lens oxygen availability. In early development, the embryonic lens receives oxygen from a network of capillaries comprised of the tunica vasculosa lentis and the anterior pupillary membrane [2]. However, as newly forming secondary lens fiber cells are just beginning to initiate the elimination of organelles and the expression of key proteins required for mature lens fiber cell formation and function, this capillary network has been eliminated leaving the lens in a hypoxic environment [2] with the vitreous and aqueous humors as the only source of oxygen for the lens [3–6]. Low levels of oxygen contained in the aqueous humor combined with consumption of oxygen by the mitochondria containing lens epithelial cells at the surface of the lens contribute to a hypoxic environment below the lens surface where lens epithelial cells differentiate into lens fiber cells [4, 5] and in the transitional zone of the lens where lens organelle degradation takes place and lens fiber cell differentiation is initiated. Consistently, studies on interior lens oxygen levels reveal a 20-fold decrease in oxygen levels from the epithelial cell-containing surface of the lens to the differentiating lens fiber cells [5]. This oxygen gradient parallels a wide-variety of hallmark events that characterize the formation of mature lens fiber cells including the elimination of cellular organelles and the expression of critical genes required for lens fiber cell transparency and function.

Consistent with a requirement for hypoxia in the elimination of organelles to form mature lens fiber cells, hypoxia has been shown to be required for induction of the mitophagy gene BNIP3L in lens fiber cells that directs the elimination of non-nuclear organelles during lens fiber cell formation [7, 8] making BNIP3L an ideal control for the present study. BNIP3L is a BH3-only member of the Bcl-2 family [9], whose expression is induced by hypoxia in other cell types [10]. BNIP3L is upregulated during reticulocyte differentiation [11], extensive studies on BNIP3L show that it is required for elimination of mitochondria during reticulocyte differentiation [12–15] and, consistently, fully mature erythrocytes in BNIP3L knockout mice retain mitochondria resulting in dysfunctional erythropoiesis and anemia [13, 16].

In addition to demonstrating a role for hypoxia in the activation of BNIP3L gene expression in newly forming secondary lens fiber cells, expression of BNIP3L was also shown to be regulated through the hypoxia-dependent binding of the transcription factor hypoxia-inducible transcription factor 1 (HIF1) to the BNIP3L promoter [8]. Consistent with the possibility that HIF1α regulates other genes needed for lens fiber cell differentiation, lenses lacking HIF1α degenerate shortly after birth and their analysis reveals extensive vacuolization and fiber cell dysmorphology consistent with aberrant lens fiber cell differentiation [17].

Hypoxia regulates all three members of the hypoxia inducible factor (HIF) family of transcription including HIF1, HIF2 and HIF3 [18–20]. In most cells, the master regulator of the hypoxic response is HIF1 [18–22]. HIF1 is the predominant HIF expressed in the lens being expressed at 120-fold higher levels than HIF2 while HIF3 is not detected in the lens [23]. Although primarily investigated as an essential regulator of vascularization [24], emerging evidence suggests that HIF1 plays key roles in the regulation of a wide variety of cellular processes required for the development, homeostasis and/or disease states of a wide variety of tissues in addition to the eye lens [25–32].

HIF1 is a heterodimer made up of two subunits, HIF1β/ARNT and HIF1α. The HIF1β/ARNT subunit is expressed constitutively but the protein level of HIF1α is tightly regulated in response to environmental oxygen levels. Under normoxic (21% oxygen) conditions, HIF1α is regulated through post-translational hydroxylation of prolines in its oxygen dependent domain (ODD) by members of the 2-oxoglutarate-dependent dioxygenase superfamily of prolyl hydroxylases (PHD1, PHD2 and PHD3) [18, 19]. Proline hydroxylation promotes interaction with the von Hippel-Lindau tumour suppressor protein, targeting HIF1α for proteasomal degradation [18, 19]. Additional hydroxylation of an asparaginyl moiety in the C-terminus inhibits binding of HIF1α to its co-factors, p300 and CREB binding protein (CBP), directly inhibiting its transcriptional activity [18, 19, 21]. Under hypoxic conditions, oxygen-dependent PHDs are inactivated, allowing HIF1α to bind to nuclear pores and translocate to the nucleus where it dimerizes with HIF1β/ARNT and recruits CBP and p300 to regulate transcription [18, 19, 21, 33].

To date, the range and spectrum of genes controlled by HIF1α, particularly in primary cells, has not been fully elucidated. In the present report we identified those lens genes regulated by HIF1α by employing Cleavage Under Targets and Release Under Nuclease (CUT&RUN) [34, 35] to map the genome-wide complement of HIF1α-DNA binding complexes present in a well-characterized primary embryonic chick lens cell model system. This model system has been previously used to successfully investigate a multiple lens properties and functions including lens cell differentiation, gene expression, autophagy/mitophagy [36, 37], PI3K signaling [38], TGFB signaling [39, 40] and gap junction expression and function [41]. Using CUT&RUN, we mapped the positions of the identified HIF1α-DNA binding complexes relative to transcription start sites of nearest neighbor genes. We then employed RNA-seq to correlate the presence of HIF1-DNA binding complexes with changes in gene expression occurring upon HIF1α-activation. Finally, we examined the relationship between HIF1α-DNA binding, gene activation or repression and chromatin accessibility through analysis of Assay for Transposase-Accessible Chromatin sequencing (ATAC-seq) data [42, 43]. For these studies, we chose CUT&RUN over Chromatin Immunoprecipitation sequencing (ChIP-seq) analysis since only a few thousand cells are required for technical success, whereas ChIP-seq requires millions of cells for the same output level [34]. Moreover, CUT&RUN assesses DNA-binding in the native state and does not require DNA-protein cross-linking, which can introduce artefacts [34].

CUT&RUN analysis using a HIF1α-specific antibody identified over 8000 HIF1α-DNA specific complexes in primary lens cells following HIF1α activation. As validation, motif analysis of the identified binding sites confirmed enrichment of the HIF1α consensus sequence. One thousand one hundred ninety of the identified binding complexes were contained in chromatin accessible regions identified by ATAC-seq [43]. Activation of HIF1α was required for activation or repression of 526 genes identified by RNA sequencing. One hundred sixteen of these genes contained HIF1α-DNA binding complexes within 10kB of the transcription start sites (TSS’s) of coding genes.

These data establish the first functional map of genomic HIF1α-DNA complexes in the eye lens. They identify HIF1α as an important regulator of a wide-variety of genes previously shown to be critical for lens formation and function and they reveal a requirement for HIF1α in the regulation of a wide-variety of multiple novel genes. Collectively, the results support a requirement for HIF1α in lens development, structure and function and they provide a basis for understanding the potential roles and requirements for HIF1α in the development, structure and function of more complex tissues.

## Results

### CUT&RUN identifies 8375 HIF1α-DNA binding complexes in the lens genome

**Fig. 1:**
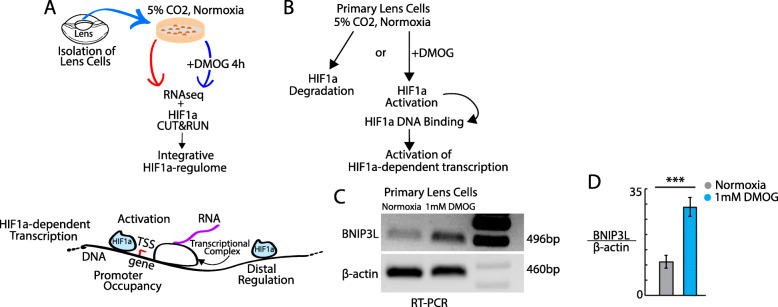
Multiomic identification of functional HIF1α DNA binding targets in the lens genome. **A** Duplicate pools of 500,000 primary chick lens cells were cultured with or without the HIF1α activator DMOG (1 mM) for 4 h. Samples were analyzed by CUT&RUN and RNA-seq analysis to identify genome-wide HIF1α-DNA binding complexes and corresponding HIF1α-dependent gene expression changes. Results were compared with ATACseq data [43] to identify the chromatin accessibility of identified HIF1α-DNA binding complexes. Integrated bioinformatics analysis was used to identify the pathways and functions specific for identified HIF1α-targets. **B** HIF1α is rapidly degraded under normoxic conditions but the presence of DMOG, inhibits prolyl hydroxylase resulting in HIF1α stabilization, DNA binding and transcriptional modulation in lens cells. **C**-**D** RT-PCR showing increased levels of the BNIP3L control transcript relative to β-actin in lens cells incubated in the presence of 1 mM DMOG for 4 h. *p < 0.05 t-test *n* = 3

Multiple lines of evidence suggest an essential role for HIF1α in regulating gene expression pathways leading to mature lens cell structure and function. The lens resides in a hypoxic environment [4–6, 44], lens deletion of HIF1α disrupts lens cell structure and development [17] and hypoxia and HIF1α are required for the elimination of non-nuclear organelles during mature lens cell formation through activation of the *BNIP3L* gene [8]. Based on these data, we hypothesized that HIF1α could regulate a wide-variety of genes involved in multiple genetic pathways important for mature lens cell structure and function. To establish the range and spectrum of lens genes regulated by HIF1α, we first sought to establish the genomic-level occupancy of HIF1α-DNA binding complexes in primary lens cells upon HIF1α activation using Cleavage Under Targets & Release Using Nuclease (CUT&RUN) (Fig. 1A-B). HIF1α was activated in primary embryonic chick (*Gallus gallus*) lens cells by exposure to 1 mM dimethyloxalylglycine (DMOG) for 4 h. This 4-h DMOG treatment was selected based on previous embryonic whole chick lens studies in *Gallus gallus* [8] showing this time of exposure resulted in robust binding of HIF1α to its cognate site of the BNIP3L gene promoter detected by ChIP-qPCR and robustly activated BNIP3L transcription detected by RT-PCR [8]. BNIP3L is an established HIF1α target in multiple cell-types [8, 45, 46]. In this study, exposure of primary lens cells to 1 mM DMOG for 4 h also resulted in a greater than 2-fold increase in *BNIP3L* mRNA (Fig. 1C-D, Supplementary Figure S1).

**Fig. 2:**
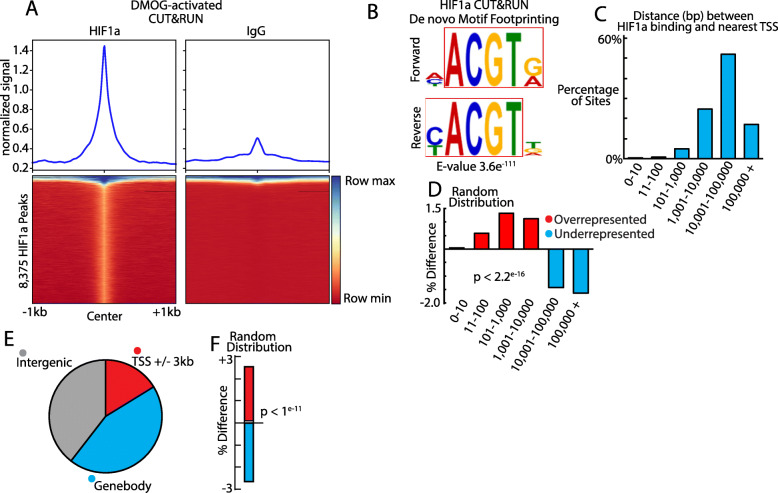
The spectrum and specificity of HIF1α-DNA binding complexes of the lens genome. **A** Heatmap of HIF1α binding signal compared to IgG signal at all genomic regions containing HIF1α-DNA binding complexes identified by CUT&RUN. **B** De novo motif analysis of HIF1α-DNA binding complexes identified by CUT&RUN. The canonical HIF1α consensus sequence was the most significantly enriched motif (E-value 3.6 × 10^− 111^). **C** Percent of the 8375 HIF1α-DNA binding complexes identified by CUT&RUN that were found within the indicated distances from the nearest gene transcription start site. **D** Relative percentage difference between the distribution of HIF1α-DNA binding complexes identified by CUT&RUN compared to computer generated HIF1α-DNA binding complexes randomly distributed across the genome and sorted by distance to the nearest gene transcription start site. (X^2^-test, *p* < 2.2 × 10^− 16^). **E** Percent of the 8375 HIF1α-DNA binding complexes identified by CUT&RUN that were found within proximal promoter regions (+/− 3kbp of gene transcription start sites), genebodies, or intergenic regions. **F** Relative percentage difference between the distribution of HIF1α-DNA binding complexes identified by CUT&RUN compared to computer generated HIF1α-DNA binding complexes randomly distributed across the genome and classified as found within proximal promoter regions (+/− 3kbp of gene transcription start sites), genebodies, or intergenic regions. (X^2^-test, *p* < 1 × 10^− 11^)

Biological duplicates of primary chicken lens cell preparations were treated with 1 mM DMOG for 4 h to activate HIF1α followed by in situ HIF1α CUT&RUN [34, 35]. Following Illumina sequencing, small chromatin fragments were mapped across the Galgal6 genome and interrogated for enrichment relative to an IgG isotype control CUT&RUN. There were 8375 HIF1α-DNA binding complexes across the Galgal6 genome with high resolution and specificity (Table S1). These exhibited specific binding within narrow regions of chromatin. A heatmap of all identified HIF1α-DNA binding complexes is shown in Fig. 2A. The data reveal the presence of a highly enriched HIF1α signal relative to the control IgG signal at genomic regions containing identified HIF1α-DNA binding complexes. To validate the binding identified by CUT&RUN, we analyzed nucleotide patterns in DNA sequences underlying genomic regions bound by HIF1α using de novo motif discovery to determine enrichment of transcription factor footprints at these loci. The most significant motif identified was the canonical HIF1α motif (5′-ACGTG-3′; E < 3.6 × 10^− 111^, Fig. 2B), confirming the presence of the HIF1α binding sequence in DNA regions identified to be bound to HIF1α by CUT&RUN. The second most significant motif identified was 5′-KCTGTR-3′; E < 8.3 × 10^− 98^ (Supplementary Figure S2) which is not known to bind HIF1α or other transcription factors.

The majority of identified HIF1α-DNA binding complexes were located within 100kbp of the nearest gene transcription start site (Fig. 2C), indicating a potential role in transcriptional regulation. Consistent with our findings, a previous study performed CHIPseq to map HIF1α binding sites in a breast cancer cell line model [47]. They found that a large majority of HIF1α binding sites were within 10 kb of the nearest transcription start site. Another study also used CHIPseq to map HIF1α binding sites in HKC-8, RCC4, and HepG2 cells [48]. They found that a majority of HIF1α binding sites were within 5 kb of the nearest transcription start site. CUT&RUN is able to identify distal transcription factor binding sites with greater accuracy and resolution compared to CHIPseq [34] and so our results likely indicate the identification of a large number of distal HIF1α binding sites that CHIPseq did not identify.

To determine whether the observed distribution could have occurred by chance, we compared binding patterns of HIF1α-DNA complexes identified by CUT&RUN on DMOG-treated primary lens cells to a computer-generated random distribution of HIF1α-DNA binding complexes across the genome. We found that the 8375 HIF1α DNA binding sites clustered closer to transcription start sites of genes compared to the computer-generated random distribution model of HIF1α DNA binding (χ^2^ test *p* < 2.× 10^− 16^, Fig. 2D). Specifically, HIF1α-DNA binding complexes were clustered more within 10 kb of transcription start sites and clustered less at ranges greater than 10 kb compared to the computer-generated model of randomly distributed HIF1α-DNA binding complexes. One thousand three hundred fifty-nine HIF1α-DNA binding complexes (16.2%) were mapped within 3 kb of transcription start sites, 3708 HIF1α-DNA binding complexes (44.3%) were found to be within gene bodies, and 3308 HIF1α-DNA binding complexes (39.5%) were mapped to intergenic regions greater than 3 kb away from transcription start sites of genes (Fig. 2E). HIF1α-DNA binding complexes were also found to be positively associated with proximal promoter regions (+/−3kbp region around transcription start sites) and negatively associated with gene body regions relative to the computer-generated random distribution of HIF1α-DNA binding complexes (*p* < 1 × 10^− 11^, Fig. 2F)). Additionally, there were 1763 gene transcription start sites that were nearest neighbors to at least two HIF1α binding sites. Three hundred ten gene transcription start sites have at least one HIF1α binding site < 3 kb AND at least one HIF1α binding site > 3 kb away.

**Fig. 3:**
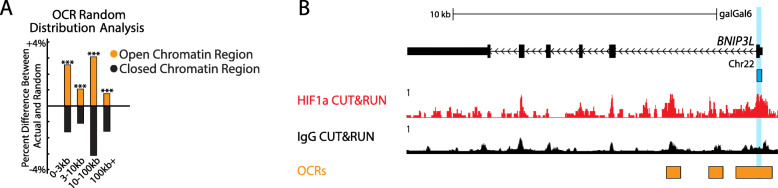
HIF1α-DNA binding complexes identified by CUT&RUN cluster in open chromatin regions. **A** HIF1α-DNA binding complexes were separated into those bound in open chromatin regions and those bound in closed chromatin regions and then sorted into distances away from the transcription start site (0-3kbp, 3-10kbp, 10-100kbp, over 100kbp). The results were compared to what is expected from the computer generated randomly distributed HIF1α-DNA binding complexes. HIF1α-DNA binding complexes were positively associated with open chromatin regions and negatively associated with closed chromatin regions at 0-3kbp, 3-10kbp, 10-100kbp, and over 100kbp distances away from the transcription start site (***p < 1 × 10^− 6^) compared to the computer-generated random distribution of HIF1α-DNA binding complexes. **B** CUT&RUN tracks of HIF1α (red) and IgG negative control (black) within the genomic region of Chr22 encompassing the *Gallus gallus* gene BNIP3L. The vertical light blue highlight and light blue box indicates a HIF1α-DNA binding complex in the proximal promoter of the gene. The orange track shows the combined chromatin accessibility map representing a combination of the differentiation stage-specific chromatin accessibility tracks obtained from [43] indicating regions of open chromatin in whole chick lens tissue

Active gene regulatory regions often cluster in regions of accessible chromatin comprised of low nucleosome density [49–51]. Notably, our previous study identified the canonical HIF1α motif as one of the top enriched motifs contained within open chromatin regions identified in the embryonic chick lens genome by ATAC-seq [43]. We therefore compared our HIF1α DNA binding complexes identified by CUT&RUN to these previously identified open chromatin regions. The analysis revealed 1190 HIF1α-DNA binding complexes (14.2%) identified by CUT&RUN that were also contained within genomic regions that exist as open chromatin regions in embryonic chick lens genome (Table S1). HIF1α-DNA binding complexes were also found to be positively associated with open chromatin regions and negatively associated with closed chromatin regions compared to the computer-generated random distribution of HIF1α-DNA binding complexes (χ^2^ test *p* < 1 × 10^− 55^). We also sought to determine if the distance between the HIF1α-DNA binding complex and the transcription start sites of genes was significantly associated with binding to open chromatin regions. We categorized the CUT&RUN-identified HIF1α-DNA binding sites by distance from the nearest gene transcription start site (0-3 kb, 3-10 kb, 10-100 kb, + 100 kb) and whether the HIF1α-DNA binding site overlapped an open chromatin region or closed chromatin region. We then repeated this categorization for a computer-generated random distribution of HIF1α-DNA binding sites and compared the differences. This analysis found that HIF1α-DNA binding complexes were positively associated with open chromatin regions and negatively associated with closed chromatin regions compared to what is expected from a computer-generated random distribution of HIF1α binding sites. This association was consistent even when separating the HIF1α binding sites by distance from the nearest gene transcription start site (Fig. 3A). These data suggest that HIF1α binding to regions near transcription start sites in the genome is biologically specific.

Such proximity to promoters and enrichment of binding within gene bodies and open chromatin regions, indicated with high probability that HIF1α is likely to be a direct transcriptional regulator of a large proportion of bound genes. Consistently, CUT&RUN identified the HIF1α-DNA binding complex previously discovered by ChIP-qPCR in the 5′-proximal upstream region of *BNIP3L* (Fig. 3B) [8] that also overlaps with open chromatin regions previously identified by ATACseq of embryonic chick lenses [43]. Additional visualizations of HIF1α CUT&RUN signal tracks and open chromatin regions in the Galgal6 genome can be found at the UCSC genome browser link (https://genome.ucsc.edu/s/jdisatha/CUTRUN_Lens_HIF1Α).

### HIF1α binding induces expression of numerous genes in lens cells

**Fig. 4:**
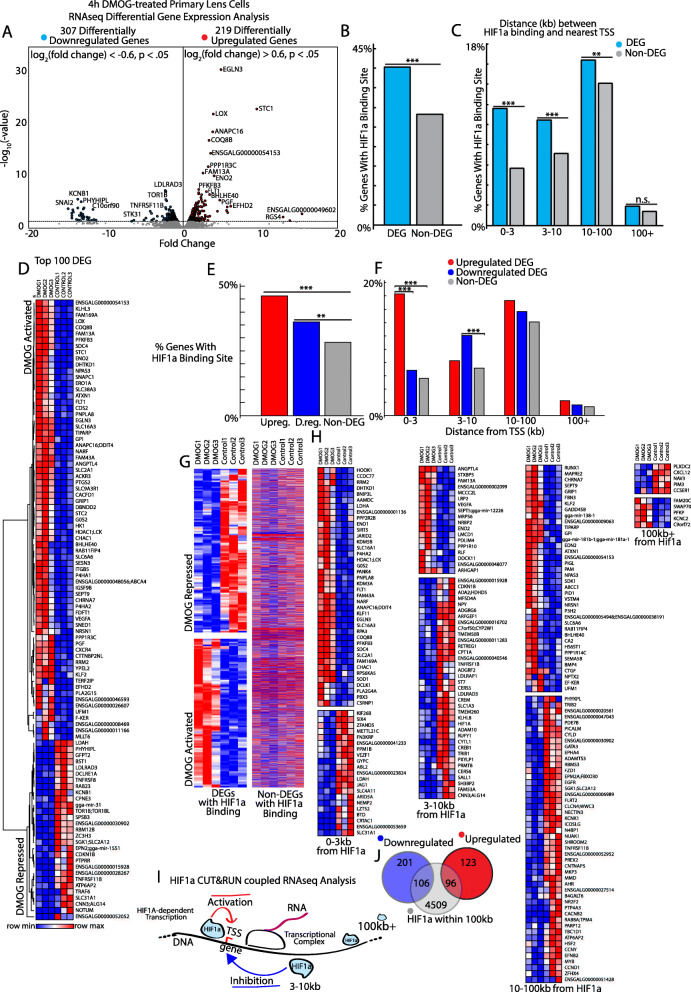
HIF1α binds to and regulates the expression of over 200 lens genes: **A** Differential gene expression analysis by RNA-seq reveals 219 upregulated genes (log2 fold change FPKM > 0.6, *p* < 0.05) and 307 downregulated genes (log2 fold change FPKM < − 0.6, *p* < 0.05) in primary lens cells treated with the HIF1α activator DMOG (1 mM) relative to control untreated cells. **B** Percent of differentially expressed genes (DEG) (|log2 fold change FPKM| > 0.6, *p* < 0.05) and non-differentially expressed genes (NON-DEG) (*p* > 0.05) that have a HIF1α-DNA binding complex. Differentially expressed genes are more significantly associated with HIF1α binding compared to non-differentially expressed genes (X^2^-test, ***p < 0.001). **C** Same analysis as (**B**) but separated by distance between the transcription start site (TSS) and the HIF1α-DNA binding complex. (X^2^-tests, ***p < 0.001, **p < 0.01, ns *p* > 0.05). **D** Heatmap of the top 100 differentially expressed genes sorted by hierarchal clustering. **E** Percent of upregulated genes, downregulated genes and NON-DEG that have a HIF1α-DNA binding complex. Upregulated and downregulated genes are significantly associated with HIF1α-DNA binding complexes. (X^2^-test, ***p < 0.001, **p < 0.01). **F** Same analysis as (**E**) but separated by distance between the transcription start site (TSS) and the HIF1α-DNA binding complex. (X^2^-test, ***p < 0.001). **(G)** Heatmap of differentially expressed (DEGs) and non-differentially expressed (Non-DEGs) genes with a HIF1α-DNA binding complex (DEGs). **H** Same as (**G**) but separated by distance from the transcription start site (0-3kbp, 3-10kbp, 10-100kbp, and over 100kbp). **I** HIF1α CUT&RUN coupled to RNA-seq analysis elucidates a significant relationship between HIF1α binding within 3kbp of the TSS and gene upregulation. In contrast, HIF1α binding between 3-10kbp of the TSS is significantly associated with gene downregulation. **J** Overlap of number of genes that are significantly upregulated or significantly downregulated and genes with HIF1α-DNA binding complexes within 100kbp of the transcription start site

To determine the range and spectrum of gene transcriptional events corresponding with HIF1α DNA binding occupancy in lens cells, we performed RNA-seq on primary lens cells under the same conditions performed for the CUT&RUN analysis. This activation of HIF1α by DMOG resulted in upregulation of 219 genes and downregulation of 309 genes (log2FC at least 0.60 in either direction at *p* < 0.05) (Fig. 4A, D, Table S2). Genes that are upregulated following treatment with DMOG have significantly lower expression levels in lens cells that were not exposed to DMOG. Genes that are downregulated following treatment with DMOG have significantly higher expression levels in lens cells that were not exposed to DMOG. To determine if there was a direct correlation between HIF1α binding and changes in gene expression, each HIF1α DNA-binding locus was mapped to the transcription start site (TSS) of nearest neighbor genes. This analysis revealed that upon HIF1α activation, 40% (212 of 528) of differentially expressed genes (DEGs) were bound by HIF1α (*p* < 0.001 χ^2^ test compared to genes unchanged in DMOG-exposed cells, non-DEG)) (Fig. 4B). We also sought to determine whether this significant association was specifically dependent on gene induction or repression. We did this by separating the differentially expressed genes into upregulated and downregulated genes. This analysis found that upon HIF1α activation, both 46.1% (101 of 219) of upregulated genes and 36.2% (111 of 309) of downregulated genes were bound by HIF1α (*p* < 0.001 and < 0.01, respectively by χ^2^ test relative to non-DEG) (Fig. 4E, G). Additionally, genes exhibiting HIF1α-DNA binding had greater magnitude fold changes (abs(log2FC), *p* < 1 × 10^− 7^ Mann-U Whitney test) compared to genes not bound by HIF1α. Taken together, these data indicated a significant role for HIF1α binding in lens cell gene regulation.

We next sought to determine whether the distance between the HIF1α-DNA binding complex and the TSS of genes was correlated with gene induction or repression in HIF1α-activated primary lens cells. A significantly higher percentage of differentially expressed genes were bound by HIF1α within 3 kb (61 of 526 (11.6%), χ^2^ test *p* < 0.001), 10 kb (55 of 526 (10.5%), χ^2^ test *p* < 0.001), and 100kbp (86 of 526 (16.3%), χ^2^ test *p* < 0.01) from the transcription start site compared to non-differentially expressed genes (940 of 16,777 (5.6%), 1191 of 16,777 (7.1%), 2366 of 16,777 (14.1%) respectively) (Fig. 4C). This indicated that proximity of HIF1α binding to the TSS correlates with activation or repression of gene transcription. Consistently, upregulated genes were significantly enriched in HIF1α-binding within 3 kb of the TSS compared to both downregulated and non-responsive genes (Fig. 4F, H). Conversely, down-regulated genes were significantly enriched in HIF1α binding between 3kbp-10kbp away from the TSS relative to non-responsive genes (Fig. 4F, H). Thus, genes induced by HIF1α were more likely to exhibit proximal HIF1α binding near the TSS and genes repressed by HIF1α were more likely to be bound by HIF1α at a greater distance from the TSS (Fig. 4I).

We also compared consensus binding sequences between HIF1α sites near induced genes versus HIF1α sites near repressed genes. Using the DREME tool, we did not find a statistically significant difference between the enriched consensus sequences at HIF1α sites near induced genes versus HIF1α sites near repressed genes.

Since there was a statistically significant association between altered expression of genes upon HIF1α activation and HIF1α binding to proximal regions within 100kbp of the transcription start site (Fig. 4C) but not at distances greater than 100kbp of the transcription start site, we defined a cutoff distance of 100kbp to mark genes whose expression levels are at least in part regulated by the activation and binding of HIF1α to proximal regulatory sites. Thus, 96 of the 219 upregulated genes and 106 of the 307 downregulated genes contain HIF1α-DNA binding complexes within 100kbp of the transcription start site (Fig. 4J).

Collectively, these data establish a significant relationship between HIF1α-DNA binding complexes to proximal gene regulatory regions and control of specific gene expression by HIF1α in lens cells. Interestingly, HIF1α acts as both an activator and suppressor of gene expression in lens cells consistent with the features of lens cell differentiation that include both activation and repression of specific genes [43, 52, 53].

### Integrated CUT&RUN and RNA-seq analysis reveals a requirement for HIF1α in the regulation of previously established as well as novel genetic pathways implicated in lens differentiation, homeostasis, structure and function

**Fig. 5:**
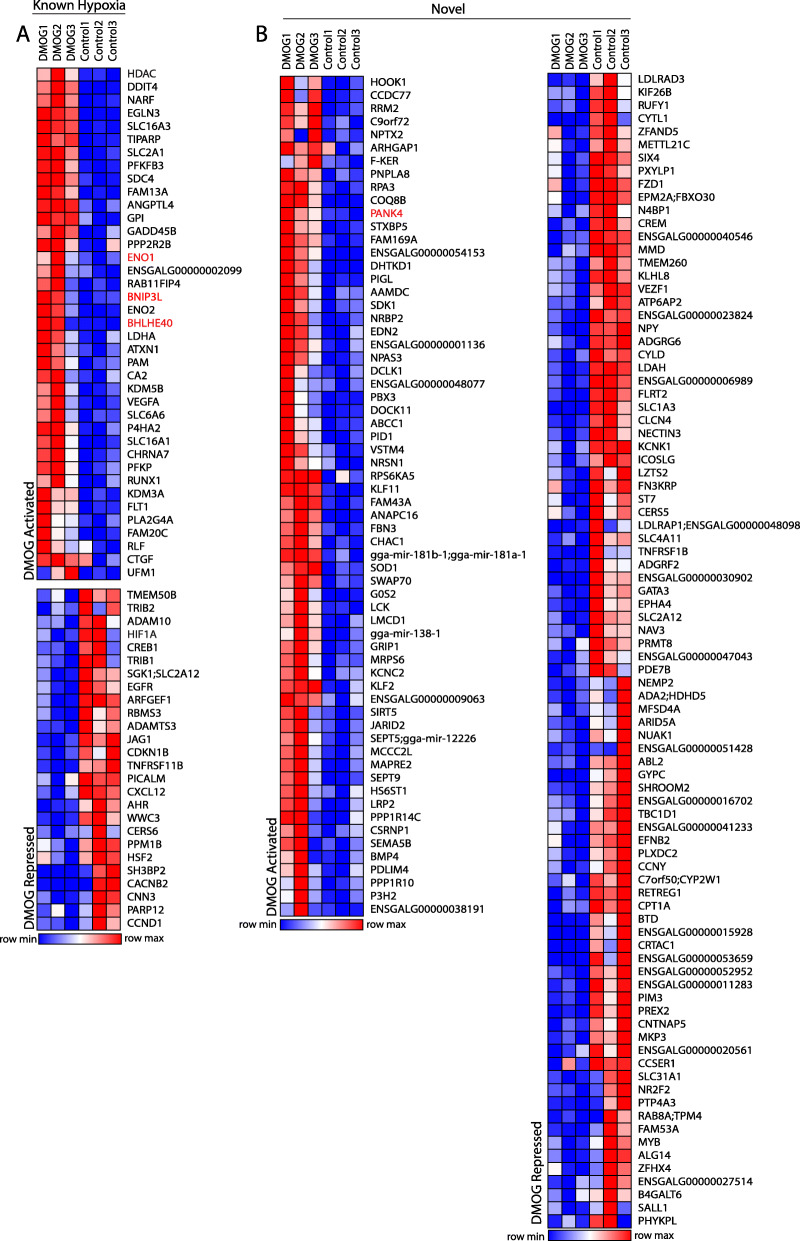
Integrated CUT&RUN and RNA-seq analysis identifies previously established and novel HIF1α-dependent lens targets. **A** Heatmap of significantly upregulated (log2FC > 0.7, *p* < 0.05) and downregulated (log2FC < − 0.7, *p* < 0.05) genes with a HIF1α-DNA binding complex that are known to be regulated by hypoxia from [57]. Level of Red indicates higher gene expression levels, and level of blue indicates lower gene expression levels. **B** Heatmap of significantly upregulated (log2FC > 0.7, *p* < 0.05) and downregulated (log2FC < − 0.7, *p* < 0.05) genes with a HIF1α-DNA binding complex that are not previously known to be regulated by hypoxia from [57]. Red indicates higher gene expression levels, and blue indicates lower gene expression levels

HIF1α and hypoxia are known to control numerous cellular processes including glycolysis, cell cycle and apoptosis [54–56]. Based on the integrated analysis of HIF1α-DNA binding complexes identified by CUT&RUN and transcriptional regulation by RNA-seq, we defined genes as HIF1α-dependent targets if they were significantly induced or repressed upon HIF1α activation and contained at least one HIF1α-DNA binding complex within 100kbp of the TSS. Employing these criteria, 202 genes were HIF1α-dependent. Of these genes, 62 were previously established as regulated by hypoxia and/or HIF1α (Fig. 5A, Table S3) in non-lens tissues [57] while 140 genes were novel (Fig. 5B, Table S3).

**Fig. 6:**
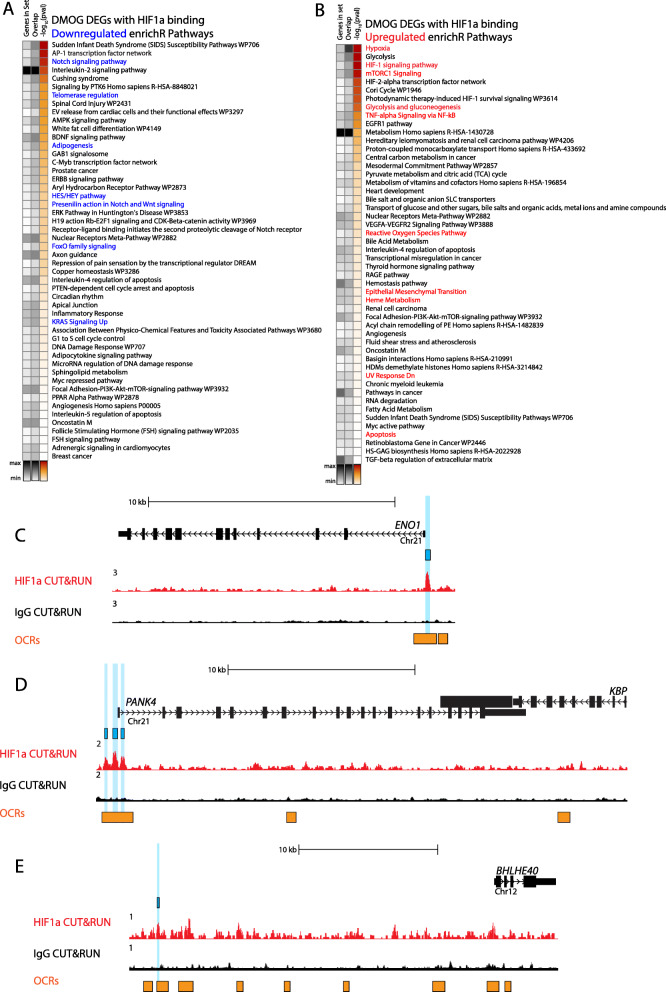
CUT&RUN and RNA-seq analysis identifies HIF1α-dependent pathways critical for lens structure, homeostasis, metabolism, and differentiation: **A** Genes with a HIF1α-DNA binding complex that are significantly downregulated (log2FC < − 0.7, *p* < 0.05) were input into Enrichr to elucidate significantly associated biological pathways and processes. The top 50 most statistically significant non-redundant pathways are shown. **B** Genes with a HIF1α-DNA binding complex that are significantly upregulated (log2FC > 0.7, *p* < 0.05) were input into Enrichr to elucidate significantly associated biological pathways and processes. The top 50 most statistically significant non-redundant pathways are shown. **C**-**E** CUT&RUN tracks of HIF1α (red) and IgG negative control (black) within the genomic region encompassing the *Gallus gallus* genes ENO1 (**C**), PANK4 (**D**), and BHLHE40 (**E**). The vertical light blue highlight and light blue box indicates a HIF1α-DNA binding complex in the proximal promoter of the gene. The orange track shows the combined chromatin accessibility map representing a combination of the differentiation stage-specific chromatin accessibility tracks obtained from [43] indicating regions of open chromatin in whole chick lens tissue

To elucidate the cellular processes and genetic pathways specific for HIF1α activation in the lens, we used Enrichr and MSigDB to identify significantly enriched processes and pathways associated with HIF1α-activated and repressed genes. Analysis of HIF1α-dependent activation pathways revealed a wide variety of previously established key lens pathways associated with lens homeostasis and maturation including activation of glycolysis [58], TNF-alpha signaling via NFKB [59, 60], reactive oxygen species pathway [36], mTORC1 signaling [37], epithelial-mesenchymal transition [61–64], heme metabolism [65, 66], UV response [58], and apoptotic pathways [67–69] (Fig. 6A and B, Table S4). By contrast, analysis of HIF1α-dependent repressed pathways revealed a wide variety of previously established key lens pathways associated with Notch signaling [70–72], Wnt signaling [73–75], and maintenance of epithelial phenotype [53, 76] (Fig. 6A and B, Table S4). The identification of these pathways suggests a novel HIF1α-dependent regulation of critical lens pathways governing lens homeostasis and maturation.

In addition to these previously established lens pathways a large number of novel pathways without previously established lens functions were also revealed. Of the upregulated genes, the novel enriched pathways included but are not limited to the IL-2/STAT5 signaling pathway while the novel repressed pathways included those involved in adipogenesis, telomerase, KRAS, and FoxO family signaling (Fig. 6A and B, Table S4).

In addition to novel pathways a wide-variety of specific HIF1α-induced genes were also identified including ENO1, PANK4, and BHLHE40. ENO1 is a multifunctional protein that is both a lens structural crystallin and is involved in glucoses metabolism [77, 78] (Fig. 6C). PANK4 has been linked to autosomal dominant congenital cataracts [79] (Fig. 6D) and BHLHE40 is a transcriptional repressor also involved in NOTCH signaling (Fig. 6E). Other HIF1α-induced genes include ANAPC16 that coordinates lens differentiation through formation of the anaphase-promoting complex [80] and BMP4 that plays a well-established role in lens development [74, 81]. Finally, the chromatin remodeling enzymes HDAC1, KDM3A KDM5A were activated by HIF1α and their induction could be important for the lens differentiation program (Table S3). Future functional analysis of these genes and pathways will likely reveal novel requirements in essential HIF1α-regulated lens processes and their identification here provides insight into their potential roles in other more complex tissues.

## Discussion

A major feature of the developing and mature eye lens is the presence of an oxygen gradient that parallels the naturally occurring surface to core differentiation of immature lens epithelial cells into mature transparent fiber cells [5]. Based on these lens features, we hypothesized that hypoxia-activation of HIF1α could regulate lens gene expression levels required to achieve the mature form and function of lens fiber cells. Consistently, a previous study showed disrupted lens structure upon lens-specific deletion of HIF1α that included dissociation of lens fiber cells, extensive lens fiber cell vacuolization and disintegration of the entire lens shortly after birth [82]. Consistent with an essential role for HIF1α in lens structure and function, we established that both hypoxia and activation of HIF1α are required for the elimination of non-nuclear organelles during lens fiber cell formation through HIF1α-dependent activation of the mitophagy protein BNIP3L [8].

Despite the previously established requirements for HIF1α in lens fiber cell organelle removal and structure, the range and spectrum of lens genetic pathways governed by HIF1α have not been identified. To identify these pathways and their sub-components, we employed a multiomics approach using CUT&RUN [34, 35] to identify and map the genomic complement of HIF1α-DNA binding complexes in the lens, RNA-seq to identify gene expression changes directly corresponding with specific HIF1α-DNA binding complexes and ATAC sequencing data to examine the chromatin accessibility of the identified genes [43].

Our analysis identified 8375 HIF1α-DNA binding complexes in the lens genome. HIF1α-DNA binding complexes were more likely to cluster near TSS compared to a computer-generated random distribution of HIF1α-DNA binding complexes (*p* < 2.2 × 10^− 16^). 2576 (30.8%). HIF1α-DNA binding complexes were mapped to regions within 10kbp of the nearest TSS. Consistent with the specificity of these HIF1α-DNA binding complexes, the HIF1α consensus sequence motif (5′-RCGTG-3′) was highly enriched in the identified HIF1α-bound genomic regions (E < 3.6 × 10^− 111^). Collectively, these results suggest an important role for HIF1α in the regulation of multiple lens pathways and genes. Consistent with HIF1α playing an important role in lens gene regulation, 1186 (14.2%) HIF1α-DNA binding complexes were localized to regions of open chromatin and the HIF1α consensus sequence motif was enriched in the identified open chromatin regions [43].

CUT&RUN has been shown to outperform traditional ChIP-seq methods in resolution, efficiency, and data quality [34]. Therefore, we chose CUT&RUN as a robust method to detect and map HIF1α-DNA binding complexes across the chick lens genome. We are confident that our CUT&RUN mapping is accurate since it replicated our previous finding using ChIP-qPCR that HIF1α binds to a 5′ proximal regulatory region of the *Gallus gallus BNIP3L* gene upon HIF1α activation [8]. Analysis of all HIF1α-DNA binding complexes indicated an enriched HIF1α signal and motif analysis of the identified HIF1α-DNA binding complexes confirmed the HIF1α binding consensus sequence as the most significantly enriched motif. To our knowledge, this study is the first CUT&RUN analysis of HIF1α.

Of the genes containing HIF1α-DNA binding complexes within 100kbp from the TSS, 96 genes were transcriptionally activated in association with HIF1α binding (log2FC > 0.6, *p* < 0.05) while 106 genes were transcriptionally repressed in association with HIF1α binding (log2FC < − 0.6, *p* < 0.05). This relationship was statistically significant for both upregulated genes (χ^2^ test *p* < 0.001) and downregulated genes (χ^2^ test *p* < 0.01). Many of the genes identified as activated by HIF1α in lens cells are associated with pathways critical for lens homeostasis, structure and transparency including glycolysis [58], TNF-alpha signaling via NFKB [59, 60], reactive oxygen species pathway [36], mTORC1 signaling [37], epithelial-mesenchymal transition [61–64], heme metabolism [65, 66], UV response [58], and apoptotic pathways [67–69].

A striking feature of the data is that an almost equal number of lens genes are repressed upon HIF1α binding suggesting that HIF1α acts as both an activator and a repressor of lens gene expression. Although many previous studies have identified HIF1α as a transcriptional repressor of individual genes [83–86], to our knowledge this is the first study to identify HIF1α as a genome-wide repressor [87].

Many of the HIF1α-repressed genes identified in this study are associated with Wnt signaling, lipid metabolism, and cell adhesion (Table S3). These pathways have previously been implicated in lens cell differentiation and homeostasis [62, 74, 75, 88–92]. This novel finding suggests a role for HIF1α-dependent repression of these pathways to regulate lens differentiation. Consistently, previous studies have shown that HIF1α represses specific genes associated with Wnt signaling in skeletal muscle and osteoblasts [93, 94] and other studies have shown that both HIF1 and HIF2 reduce expression of enzymes needed for fatty acid breakdown in lipid metabolism [95].

In addition to establishing HIF1α as both an activator and a repressor in lens cells, our analysis also identified a novel association between gene regulation and the distance of HIF1α-DNA binding complexes relative to TSS’s of activated or repressed genes. Specifically, HIF1α binding to regions within 3kbp of gene TSS’s is significantly associated with gene activation. Conversely, HIF1α binding to more distal regions between 3kbp-10kbp of gene TSS’s is significantly associated with gene repression. This difference has implications for the mechanisms underlying HIF1α control of gene expression in the lens and likely a wide-variety of other tissues. It is possible that HIF1α-DNA binding complexes within 3kbp of TSS’s promote open complex formation through interactions with other transcription factors and transcriptional co-activators that are required for HIF1α activation including P300, CBP, and HIF1β [18, 19, 21]. Conversely, distant HIF1α-DNA binding complexes more than 3kbp from TSS’s may repress open complex formation of gene expression by recruiting these transcriptional regulators away from TSS’s. These possibilities are consistent with previous studies linking the action of transcription factors with their binding relative to the TSS whether proximal or distal [96, 97]. To our knowledge, the present study is the first to-date to find a statistically significant association between genome wide HIF1α binding site distances and activation or repression of multiple genes.

Many of the identified HIF1α-dependent genes also have well established functions critical for lens cell metabolism, structure, transparency, differentiation, and development. An autosomal dominant mutation in the identified HIF1α-activated PANK4 gene is associated with congenital posterior cataract [79] and is therefore critical for lens transparency. The HIF1α-activated ENO1 gene (tau crystallin) is both a lens structural protein and a glycolytic enzyme [77]. BNIP3L, BMP4, GATA3, CDKN1B, HES5, JAG1, and VEGFA all play important roles in lens cell development and differentiation (Table S3) [8, 70, 74, 81, 98–105]. Therefore, this study suggests that activation of HIF1α in the lens plays a crucial role in maintaining these important lens-specific functions.

Many of the identified HIF1α-dependent genes also have well established functions critical for mitochondria biogenesis, structure, function, metabolism, and homeostasis (Table S5). The HIF1α-induced gene BNIP3L is required for elimination of mitochondria, endoplasmic reticulum, and golgi apparatus during lens fiber cell differentiation [7, 8]. SOD1 was found to be induced, it is involved in protection against reactive oxygen species in lens cells [106, 107] and plays a role in mitophagy during myoblast differentiation [108]. SIRT5, is involved in regulation of acetylation of the lens chaperone protein α-crystallin [109] and regulates acylation of mitochondrial proteins involved in the activation of mitochondrial function in adipose-derived mesenchymal stem cells [110]. Therefore, this study suggests that activation of HIF1α in the lens is critical for the regulation of several mitochondria-associated functional proteins that control mitophagy, protection from reactive oxygen species, and mitochondrial biogenesis.

This study focused on responses of primary lens cells to a chemical activator of HIF1α. However, gene expression in the actual lens is complicated by regional differences in gene expression and the hypoxic gradient of the lens itself. Therefore, further analysis of the gene expression patterns detected in the present study and the role of HIF1α in their regulation will need to be evaluated in future whole lens analysis. Nevertheless, we are confident that the majority of genes detected will have functional consequences in the lens and consistently the BNIP3L gene that has been shown to be expressed in lens fibers to initiate organelle degradation upon binding of HIF1α [8] was found in the present study to both exhibit activated expression and binding of HIF1α.

Additionally, the data also identify the HIF1α gene as a significantly repressed gene upon HIF1α activation. This has also been observed in other model systems [111, 112] and likely represents a negative feedback loop to acutely regulate the expression of genes by HIF1α. In the eye lens, this might be especially important for HIF1α-mediated regulation of lens fiber cell remodeling and maturation.

In addition to identifying known HIF1α-dependent genes, the present analysis also identified 151 novel HIF1α-dependent genes. These include but are not limited to *JARID2, BMP4, SIX4, GATA3, ARID5A.* These genes are involved in metabolism, chromatin remodeling, development and differentiation of a variety of tissues and cell types (Table S3). Their identification suggests novel HIF1α-dependent roles of the genes in lens and other tissues and suggest novel functions for HIF1α.

Although 202 genes showed a direct relationship between binding of HIF1α and gene activation or repression, an additional 324 genes exhibited activated or repressed gene expression upon HIF1α activation in the absence HIF1α binding. These results suggest that HIF1α acts to regulate downstream events that control expression of these genes including downstream transcription factors or chromatin remodeling proteins encoded by *BHLHE40, HDAC1, JARID2, KDM3A, and KDM5B* (Table S3) detected in the present report and previously established to be regulated by HIF1α [57]. These downstream factors could activate or repress expression of these genes by altering chromatin accessibility.

Four thousand five hundred nine genes that exhibited HIF1α binding did not exhibit altered expression levels even though they were the nearest neighbor to HIF1α binding sites extending out to 100kbp away from cognate TSS’s. This is not uncommon, as many previous studies have found that not all transcription factor binding sites are associated with active changes in gene expression [97, 113] and it is possible that activation or repression of these genes by HIF1α is dependent on the presence of additional transcription factors and/or co-activators/co-repressors not present or active under the conditions of the present study. Alternatively, the data could indicate a role for HIF1α in chromatin structure and/or maintenance and future studies will be needed to address these possibilities.

Although this paper specifically focuses on binding and activation of HIF1α, the hypoxic environment of the lens is likely to activate additional hypoxia-regulated processes via other factors [18]. For example p53 that is hypoxia-regulated is known to be involved in HSF4-dependent regulation of lens fiber cell differentiation [114]. Further analysis will be required to identify additional hypoxia-regulated lens transcriptional activators and functional pathways. Indeed in the present study, 519 of the 5071 (10.23%) non-DEGs with HIF1α binding sites have known associations with hypoxia and it is possible that these genes might be regulated by hypoxia but not HIF1α.

## Conclusions

Collectively, the present report establishes a functional genomic map of novel HIF1α-regulated genes of the eye lens and supports the hypothesis that hypoxia and HIF1α-dependent gene regulation is critical towards achieving mature lens structure and transparency. The data also establish HIF1α as both an activator and repressor of lens gene expression, reveal novel HIF1α-DNA complex features, confirm previously established HIF1α targets and identify novel HIF1α targets that likely are important for the formation and function of the lens and possibly other tissues.

## Methods

### Primary lens cell culture

Primary chick lens epithelial cell cultures were prepared from the lenses of Embryonic day 10 (E10) White Leghorn embryonated chicken eggs (Charles River Laboratories, Storrs, CT) as previously described [36]. Briefly, primary lens cells were isolated from chicken lenses by trypsinization and agitation. Cells were plated onto dishes coated with mouse laminin (Invitrogen, prod no: 23017–015) and cultured in Medium 199 (Invitrogen, prod no: 11150) supplemented with 10% FBS (Invitrogen, prod no: 16140–071) and penicillin-streptomycin antibiotic mix (50 units/ml; Invitrogen, prod no: 15140–122). The resulting primary lens cells were incubated for 2 days at 37 °C in the presence of 5% CO_2_ before treatment with DMOG as described below.

### Analysis of BNIP3L gene expression in chick lens epithelial cell culture

BNIP3L transcript levels were evaluated in RNA isolated from 100,000 primary lens epithelial cells. BNIP3L transcript levels were measured by RT-PCR using the SuperScript® III one-step RT-PCR system with Platinum Taq polymerase (Invitrogen) according to the manufacturer’s instructions with β-actin as control. 100 ng of total RNA was assayed following isolation from lens epithelial cells treated or not for 4 h with 1 mM DMOG. BNIP3L transcripts were amplified for 25 PCR cycles with a 54 °C annealing temperature and the primer sequences: Forward primer – GCAATGGCAATAGCAATGAT and reverse primer – ATGTAGATGCCTAGTCCCAA. β-actin transcripts were amplified for 25 PCR cycles with a 58 °C annealing temperature and the primers sequences: Forward primer – AGCCATCTTTCTTGGGTATGGA and reverse primer – AATCCTGAGTCAAGCGCCAA. The RT-PCR conditions employed were as described in [8]. Conditions were optimized for the amount of RNA used and the number of cycles employed to ensure linear amplification. Statistical significance was determined by student’s t-test.

### CUT&RUN on DMOG-treated lens cells and bioinformatics analysis

Primary lens cells were treated with the HIF1α activator dimethyloxalllyl glycine (DMOG, Santa Cruz Biotechnology, prod no: sc200755) (1 mM) for 4 h at 37 °C. DMOG is a cell permeable competitive inhibitor of prolyl hydroxylase domain-containing proteins such as the PHDs. DMOG has been shown to activate HIF1α, under normoxic (21% O_2_) conditions in a wide variety of cell types [115, 116] including lens cells [8]. DMOG is a widely employed activator of HIF1α through its ability to inhibit prolyl hydroxylase enzymes resulting in the prevention of HIF1α degradation through the proteasomal pathway in the presence of oxygen [115, 116]. Since DMOG inhibits prolyl hydroxylases that could be involved in the regulation and degradation of other lens proteins, it is possible that DMOG could influence other lens functions in addition to activation of HIF1α. However, since this study focused on altered gene expression directly linked with the presence of DMOG dependent HIF1α-DNA complexes we are confident that our results are at least specific for HIF1α DNA binding and are unlikely to result from HIF1α-independent effects of DMOG that might occur in lens cells.

Cleavage Under Targets & Release Using Nuclease (CUT&RUN) [34, 35] was performed on unfixed permeabilized primary lens cells incubated with a HIF1α-specific ChIP grade antibody (Abcam, ab2185) and an IgG negative control antibody (Invitrogen, prod no: 10500c) to elucidate genome-wide HIF1α-DNA binding complexes. The protocol was adapted from Meers et al., 2019 [117]. Briefly, primary lens cells were harvested, counted, and pooled into biological duplicates of 500,000 cells each and resuspended in 1.5 ml Wash Buffer without Digitonin (20 mM HEPES pH 7.5, 150 mM NaCl, 0.5 mM Spermidine, Protease Inhibitor). After three rounds of washing and centrifuging for 3 min at 600 g, the resuspended cells were washed in 1 ml wash buffer and ConA-coated magnetic beads (Bangs Labroatories, prod no: BP531, 10 μl per sample) were added followed by gentle rotation at room temperature for 10 min. Each sample was divided into two equal volumes and the supernatants removed using a magnet stand. 150 μl of antibody buffer with 0.1% final concentration digitonin was added to each aliquot with gentle agitation to permeabilize the cells. For each biological sample, one aliquot of ConA-bound cells was incubated with ChIP-grade HIF1α antibody and the other aliquot was incubated with IgG antibody at a 1:100 dilution and rotated at 4 °C overnight. HIF1α antibody specificity was confirmed by western analysis of primary lens cell nuclear extract and by ChIP-qPCR published in a previous study [8]. Antibody-bound ConA-bound cells were washed twice with 1 ml wash buffer with 0.1% digitonin. The supernatant was removed and 150 μl of Protein A-MNase (700 ng/mL final concentration, Addgene #123461) [117] in wash buffer with digitonin was added to the pellet with gentle agitation and the mix rotated at 4 °C for 1 h. Cells were washed twice with 1 ml of digitonin wash buffer and once with 1 ml low salt rinse buffer (20 mM HEPES pH 7.5, 0.5 mM Spermidine, 0.05% Digitonin). The supernatant was removed and 200 μl of ice-cold incubation buffer (3.5 mM HEPES pH 7.5, 10 mM CaCl2, 0.05% Digitonin) added with gentle agitation. The resulting samples were incubated on ice for 5 min before removing the supernatant using the magnet stand followed by the addition of 200 μl STOP buffer (170 mM NaCl, 20 mM EGTA, 0.05% Digitonin, 50μg/mL RNAse A, 25 μg/mL Glycogen) with gentle agitation. The samples were incubated for 30 min at 37 °C to release CUT&RUN fragments. Following incubation, the supernatant containing CUT&RUN fragments was transferred to a fresh microcentrifuge tube. CUT&RUN fragments were treated with 2 μl 10% SDS and 2.5 μl Proteinase K (20 mg/ml) for 1 h at 50 °C. DNA was isolated by standard Phenol-Chloroform extraction using Qiagen MaXtract tubes, and the DNA pellet resuspended in 30 μl 1 mM Tris-HCl pH 8 0.1 mM EDTA. Library prep was carried out using the NEBNext Ultra II DNA Library Prep Kit for Illumina (NEB E7645) following the manufacturer’s instructions [118]. Samples were resuspended in 15 μl of 0.1x TE Buffer. Libraries were pooled and PE50 sequencing on an Illumina Hiseq platform was performed.

Reads were trimmed with Trimmomatic [119] with the following settings (ILLUMINACLIP: 2:15:4:4:true SLIDINGWINDOW:4:15 MINLEN:25) and aligned to the galgal6 genome (ensemble version 96) using Bowtie2 [120] with the following settings: (−-end-to-end,--dove-tail,--phred33). Samtools [121] was used to retain fragments < 120 bp, and to remove reads in mitochondrial DNA and remove reads overlapping low-complexity regions in the Galgal6 genome as defined by the RepeatMasker [122] track in the UCSC genome browser. HIF1α-DNA binding complexes were identified using MACS2 [123, 124] by pooling the HIF1α BAM files versus the pooled IgG BAM files using the following settings: (narrowPeak setting, q value cutoff 0.05, −-SPMR, −-keep-dup auto). MACS2 on CUT&RUN samples identified 8375 HIF1α-DNA binding complexes in the primary chick lens cells (Table S1). Although some studies have detected low levels of HIF1α-DNA binding under normoxic conditions in non-lens tissues [125], we have previously shown that HIF1α is barely detectable in lens cells in the absence of hypoxia or DMOG activation [8].

### RNA-seq on DMOG-treated lens cells and bioinformatics analysis

Biological triplicates of primary chick lens cells were incubated in the presence or absence of 1 mM DMOG for 4 h at 37 °C. Total RNA was isolated from primary lens cells using TRIZOL® reagent (Invitrogen, prod no: 15593018) according to the manufacturer’s instructions. Libraries were prepared following Illumina’s TruSeq-stranded-total-RNA-sample preparation protocol. Paired-end sequencing was performed on Illumina’s NovaSeq 6000 platform. Cutadapt [126] was used to remove adaptor-contaminated reads, low quality bases and undetermined bases. Reads were mapped to galgal6 genome (ensemble version 96) using Bowtie2 [120] and HISAT2 [127]. The mapped reads were assembled using StringTie [128]. Comprehensive transcriptomes were generated using gffcompare and StringTie [128]. The R package edgeR [129] was used to estimate the expression levels of all transcripts and to identify differentially expressed genes (log2 fold change FPKM > 0.6 or < − 0.6) with parametric F-test comparing nested linear models (*p* < 0.05). Five hundred twenty-six genes were found to be differentially expressed in DMOG-treated primary lens cells relative to untreated primary lens cell samples (Table S2).

### Additional bioinformatics methods

MACS2 generated bedgraph files for the HIF1α and IgG CUT&RUN samples were converted to BIGWIG format and visualized in UCSC Genome Browser (https://genome.ucsc.edu/s/jdisatha/CUTRUN_Lens_HIF1Α) [130]. Genomic coordinates of chick lens open chromatin regions mapped by ATAC sequencing [43] were lifted to galgal6 using UCSC Genome Browser liftover tool. HIF1α-DNA binding complexes identified by CUT&RUN that are also contained within previously identified open chromatin regions [43] are noted in Table S1.

The Bedtools [131] Shufflebed function was used to generate a negative control background of HIF1α-DNA binding complexes randomly distributed across the galgal6 genome using default settings.

The ChIPseeker [132], bedtools [131] and Closestbed tools were used to identify relative distances of HIF1α-DNA binding complexes to the nearest gene transcription start sites (Table S1).

Deeptools [133], plotProfile and plotHeatmap functions default settings were used to generate the profile and heatmap in Fig. 2A. Each site identified as a HIF1α binding site by the MACS2 bioinformatics tool is overlayed in a window +/− 1000 bp around the peak of the binding site. The heatmap and profile plot generated by deepTools indicate the signal strength of the HIF1α CUTandRUN signal and compares it to the IgG negative control signal across all of the identified HIF1α binding regions.

MEME-suite [134] DREME tool [135] default settings were used to identify short-ungapped overrepresented sequence patterns in the HIF1α-DNA binding complexes at genomic regions identified by CUT&RUN.

HIF1α-dependent genes identified by our multiomic analysis were cross-referenced with hypoxiadb [57] to elucidate previously known hypoxia-dependent genes compared to novel HIF1α-dependent genes.

Enrichr [136–138] and Msigdb [139, 140] tools were used to identify significantly enriched processes and pathways associated with the identified HIF1α-dependent genes.

All heatmaps shown were generated with the Morpheus Bioinformatics tool (https://software.broadinstitute.org/morpheus/).

### Statistical analysis

Chi-squared goodness-of-fit test was performed in R version 3.6.3 using the chisq.test() function to test the null hypothesis that the distribution of HIF1α binding sites were similar to what is expected from a random distribution of sites generated by bedtools ShuffleBed. (Figs. 2D and F and 3A). All other chi-square tests were performed to test against the null hypothesis of no significant differences in number of genes with HIF1α binding sites categorized in various different ways (Fig. 4B, C, E, F). Bonferroni corrected *p*-values are reported.

## Supplementary Information

**Additional file 1: Table S1**. HIF1α binding peaks enriched by CUT&RUN and detected with MACS2 at q < 0.05 in DMOG-treated primary lens cells.

**Additional file 2 Table S2.** Gene expression analysis via RNA-seq of DMOG-treated vs untreated primary lens cells.

**Additional file 3 Table S3.** Gene ontologies, pathways, and select literature references for differentially expressed genes with HIF1α binding.

**Additional file 4: Table S4.** Enriched gene ontologies and pathways associated with HIF1α-bound upregulated and downregulated genes as determined by MsigDB and Enrichr.

**Additional file 5: Table S5.** HIF1α-regulated genes with associated mitochondria functions.

**Additional file 6: Figure S1.** Full length version of the RT-PCR agarose gel from Fig. 1C.

**Additional file 7: Figure S2.** Second most statistically significant enriched motif in HIF1α-DNA binding regions identified by CUT&RUN.

## Data Availability

Raw sequencing reads for the CUT&RUN experiment have been deposited at NCBI-GEO series accession number GSE166626. Raw sequencing reads for the RNA-seq experiment have been deposited at NCBI-GEO series accession number GSE166632. ATACseq data were obtained from supplemental files from [31].

## Abbreviations

CUT&RUN: Cleavage Under Targets and Release Under Nuclease
ATACseq: Assay for Transposase-Accessible Chromatin sequencing
HIF1α: Hypoxia inducible factor 1 alpha
TSS: Transcription start site
ChIP-qPCR: Chromatin immunoprecipitation coupled with quantitative polymerase chain reaction
FPKM: Fragments per kilobase of exon per million fragments mapped
DMOG: Dimethyloxalylglycine
