# Supplementary figures and images for: A functional map of genomic HIF1α-DNA complexes in the eye lens revealed through multiomics analysis
Source: BMC Genomics. 2021 Jul 3;22:497. doi: 10.1186/s12864-021-07795-9 (PMC8254356; doi:10.1186/s12864-021-07795-9)

## Supplementary Figure S1

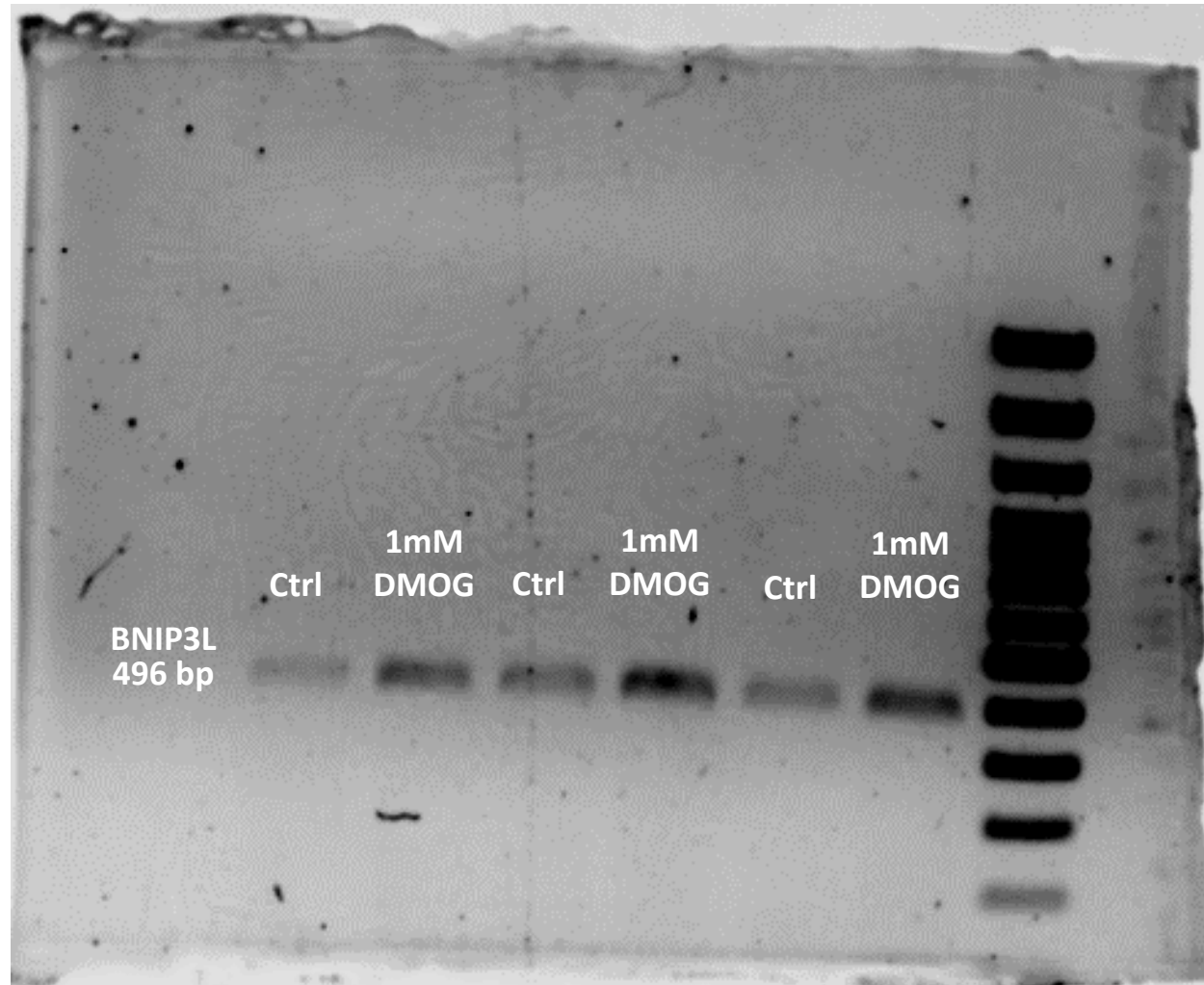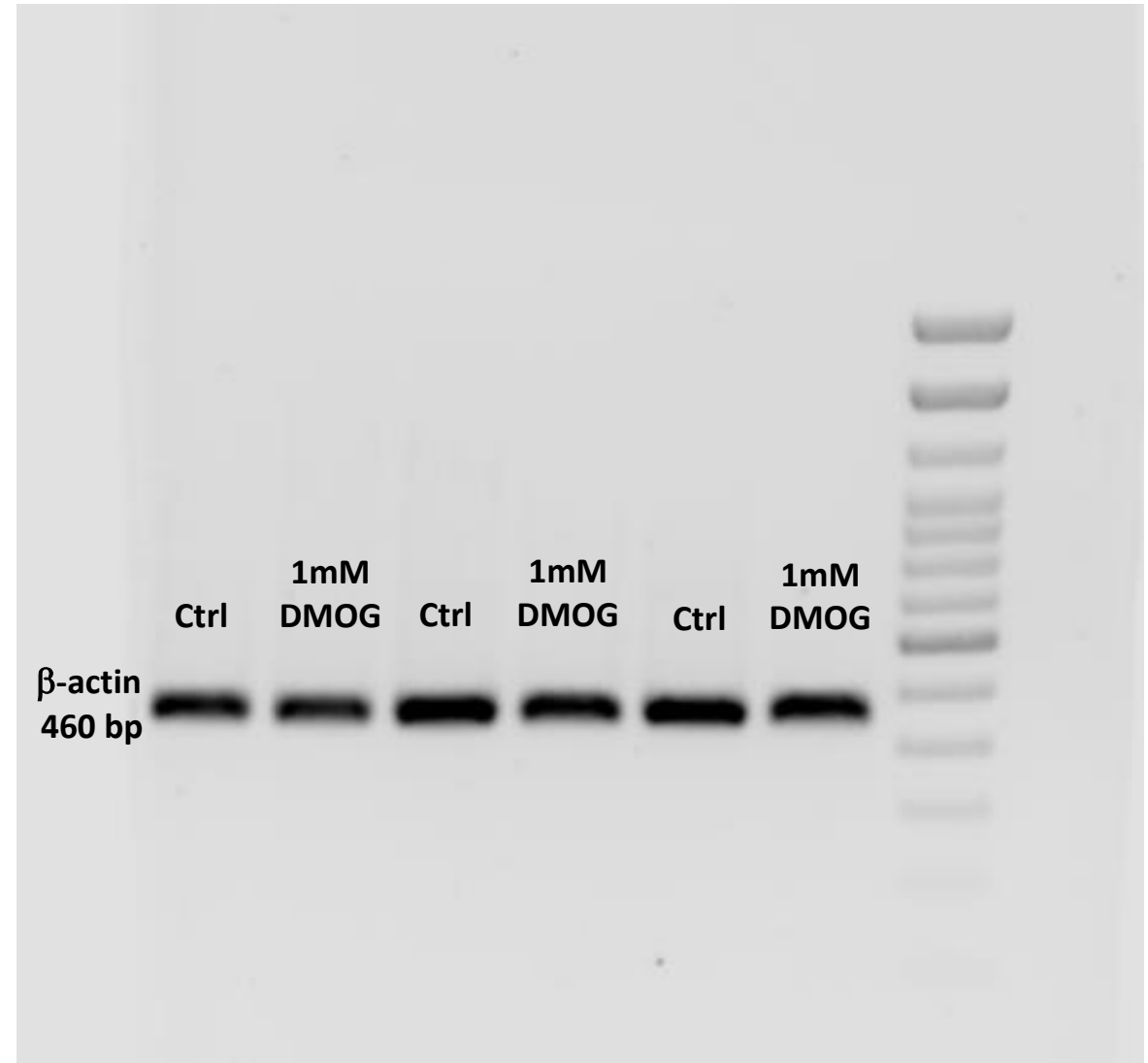

Supplement: Supplementary file 6 — Additional file 6: Figure S1. Full length version of the RT-PCR agarose gel from Fig. 1C. [file 12864_2021_7795_MOESM6_ESM.pdf]

Supplementary Figure S2

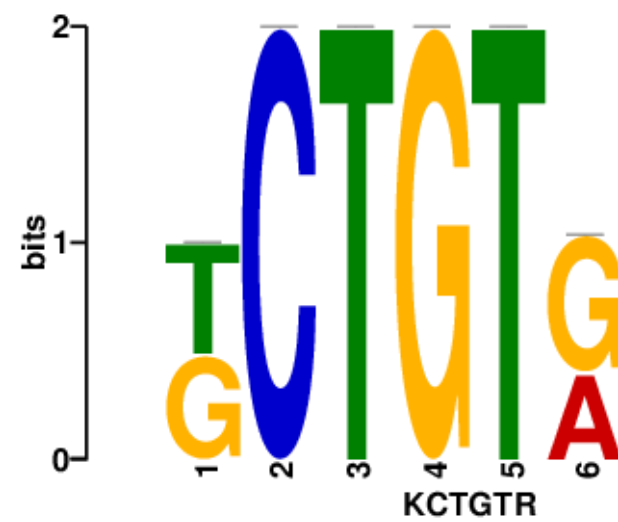

E-value  $8.3e^{-98}$

Supplement: Supplementary file 7 — Additional file 7: Figure S2. Second most statistically significant enriched motif in HIF1α-DNA binding regions identified by CUT&RUN. [file 12864_2021_7795_MOESM7_ESM.pdf]
